# Assessment of plasma BMP-2, BMP-7, BMP-10, vitamin D, and TGF β1 in simple fractures among Sudanese patients

**DOI:** 10.1371/journal.pone.0247472

**Published:** 2021-02-19

**Authors:** Amin Ahmed Ali, Muawia Mohamed Mukhtar, Samir Shaheen, Abdelrahim Osman Mohamed

**Affiliations:** 1 Department of Biochemistry, Faculty of Medicine–University of Khartoum, Khartoum, Sudan; 2 Department of Immunology at Institute of Endemic Diseases, University of Khartoum, Khartoum, Sudan; 3 Department of Orthopedics and Trauma surgery, Faculty of Medicine–University of Khartoum, Khartoum, Sudan; Assiut University Faculty of Medicine, EGYPT

## Abstract

**Background:**

Bone morphogenetic proteins (BMP) are multifunctional proteins. They work as cytokines regulating osteogenesis during fracture healing process. The objectives of this study were to assess changes in BMPs during fracture and their correlations to Fracture’s healing.

**Methods:**

Case-Control hospital–based study conducted from January 2018 to January 2019. Demographic data, anthropometric measurements, and blood samples were collected from patients and controls (18–65 years old). Plasma concentrations of selected BMPs and vitamin D were measured using quantitative enzyme linked immunosorbent assay (ELISA). SPSS version 25 was used to calculate frequencies, Pearson correlation tests, chi-square and unpaired t-test.

**Results:**

Sixty-five patients with fractures and Sixty-five controls were studied. Means of plasma concentrations were (TGFβ1 = 21.07 ng/ml ±8.49 and 19.8 ng/ml ±7.2) (BMP-2 = 76.3 pg/ml ± 156.6 and 55.5 ng/ml ± 127.9) (BMP-7 = 13.02 pg/ml ±43.5 and 64.6pg/ml ±250) (BMP-10 = 8.14 pg/ml ±12.7 and 5.48 pg/ml ±11.3) (Vitamin D mean was 24.94 ng/ml ±13.2 and 26.2 ng/ml ±11.6) in patients and controls, respectively. Forty-five subjects were enrolled into follow up study: 30 males, 15 females. Healing time mean was 4.13± 2.6 months. No significant correlation between BMP-2/BMP-7 with healing time.

**Conclusions:**

BMP-7 was significantly lowers in the plasma of patients that controls (P = 0.042). Low Vitamin D was observed among Sudanese participants.

## Introduction

Bone morphogenetic proteins (BMPs) are multifunctional proteins secreted by macrophages and assist in bone development and fractures’ repair [1]. Twenty subtypes of BMPs were introduced and ordered according to their biological functions [2]. BMPs are growth factors that help generation of new osteocytes (osteoinductive). This is carried out by activation of specific membrane bound receptors [3].

BMP-2 and BMP-7 were found to be responsible for recruiting mesenchymal stem cells. BMP-2 functions as a stimulant of mesenchymal cells differentiation into osteoblasts [4]. Recent studies support the use of BMP-2 and BMP-7 in clinical trials [5], while others reported that BMP-2 and BMP-7 might not have a role on fracture healing [6].

Many factors affect healing of fractures; such as gender [7], age [8], body mass index (BMI) [9], race [10], nutritional status [11], drinking black tea [12], and occupation [13]. Tobacco smoking and alcohol are well known for their harmful effect on fractures’ healing [14, 15].

Studying the pathophysiology of BMPs during fracture before healing might help to challenge reports that bone morphogenetic proteins are not useful in treating fractures [16], and helps better understanding of its need for future clinical uses for all long bones and not tibia fracture alone [16]. The aim of this study was to investigate whether plasma bone morphogenetic proteins change after fracture, when compared with matched healthy controls.

## Materials and methods

This is a Case-Control study. The study was conducted on the five main hospitals providing orthopedic surgery care in Khartoum state, Sudan. Duration from January 2018 to January 2019. Sixty-five patients with fractures and sixty-five healthy controls were included in this study. Inclusion criteria: All healthy individuals with no comorbidities in the patients group and control group, males and females from 18 to 65 years old. In the patients group, including subjects with closed long bone fractures, while exclusion criteria were for those with comorbidities or bone disease and those who smoke tobacco or drink alcohol.

After taking demographic data and anthropometric measurements, blood samples were taken (6 ml) into heparin and EDTA Vacutainers. Blood samples from were taken from each patient once as they have presented in variable time since fracture. Occupation was classified based on physical activities into: high, intermediate, and low physical activities jobs [17]. Black tea drinking was classified based on previous study [12]. Hemoglobin concentration was measured using a standard method. Plasma was separated and stored at -80°C freezer to be used for quantitative enzyme linked immunosorbent assay (ELISA). BMP-10 served as a negative control, while TGFβ1 as a positive control.

Quantitative ELISA: Using prepared ELISA kits for 25-OH Vitamin D from (EUROIMMUN, Germany), BMP-2, BMP-7, BMP-10, and TGFβ1 from R&D systems (R&D systems MA, USA)–following manufacturer’s protocol [18, 19].

Long bone fracture healing follow-up: A series of clinical examinations and radiographic images were conducted at specified follow up sessions every 2 weeks including plain X-ray and Physical examination at the orthopedic surgery clinic. Follow up of patients up to point of regaining functional recovery i.e. For the upper limb function in daily activities such as combing, bathing, lifting, and taking care of hygiene, while for the lower limb; standing, walking unassisted, climbing stairs, and squatting. The orthopedic surgery team for each hospital conducts follow up until reaching optimum physical composite score (PCS) [20].

### Data analysis

Done using software platform offers advanced statistical analysis SPSS (version 25) (IBM Corp., Armonk, N.Y., USA). Data were expressed as: mean (m) and standard deviation (SD). Correlation (Pearson and Spearman), Chi-square and unpaired t-test were performed. P value < 0.05 was considered statistically significant.

### Ethics approval and consent to participate

Approved by the University of Khartoum Research Board (Professor Ammar A. Tahir, Dr. Sulafa K. M. Ali, Dr. Siham A. Balla) and University of Khartoum Research Ethical Committee (Professor Sheickh Mahgoub, Dr. Shaza K. Abbas, Professor Kamal Zaki). Approved by the Ministry of Health Khartoum State Research and Ethics Committee (Professor Mohamed A. A. Kareem, Dr. Sawsan M. Osman). Approved by selected hospitals. Written consent was used.

## Results

Subjects were aged from 18 to 65 years (mean = 37.2 years ±12.4 for patients and 37.2 years ±12.4 for controls). Patients were 68% males and 32% females, while Controls were 69% males and 31% females **(Table 1)**. Both patients and control groups shared similar demographic features, with no significant differences **(Table 1)**. BMP-2 plasma concentrations were 76.3 pg/ml ± 156.6 in patients and 55.5 pg/ml ± 127.9 in controls, BMP-7 plasma concentrations were 13.02 pg/ml ±43.5 in patients and 64.6pg/ml ±250 in control **(Table 2)**. Vitamin D concentrations were 24.94 ng/ml ±13.2 in patients and 26.2 ng/ml ±11.6 in controls **(Table 2)**.

**Table 1:** Demographic data of patients group and control group.

Variable	Patients	Control	unpaired t-test*
Age (m±SD)	37.2 years ±12.4	37.2 years ±12.4	t = 0.00
≤ 40 years	38 (58.5%)	41 (63.1%)	p = 1.0
> 40 years	27 (41.5%)	24 (36.9%)	
Gender Males	44 (68%)	45 (69%)	t = 0.00
Females	21 (32%)	20 (31%)	p = 1.0
BMI (m±SD)	22±4 kg/m^2^	23±4.8 kg/m^2^	t = 0.00
<18 kg/m^2^	7 (10.8%)	5 (7.7%)	p = 1.0
18–25 kg/m^2^	32 (49.2%)	32 (49.2%)	
25–30 kg/m^2^	22 (33.8%)	26 (40%)	
> 30 kg/m^2^	4 (6.2%)	2 (3.1%)	
Occupation			
Manual Labor	19 (29%)	18 (28%)	t = 0.00
Mental Labor	46 (71%)	47 (72%)	p = 1.0

* p>0.05 indicates no significant difference between case and control groups

BMI: body mass index, m: mean, SD: standard deviation.

**Table 2:** Mean (m), Standard deviation (SD), of concentrations in patients and controls.

Variables	Patients (m±SD)	Control (m±SD)	
**Hemoglobin**	14.5±2.5 g/dl	15.6±1.9 g/dl	p = 0.004*
**Vitamin D**	24.94 ng/ml ±13.2	26.2 ng/ml ±11.6	p = 0.564
**TGFβ1**	21.07 ng/ml ±8.49	19.8 ng/ml ±7.2	p = 0.084
**BMP-2**	76.3 pg/ml ± 156.6	55.5 pg/ml ± 127.9	p = 0.718
**BMP-7**	13.02 pg/ml ±43.5	64.6pg/ml ±250	p = 0.042*
**BMP-10**	8.14 pg/ml ±12.7	5.48 pg/ml ±11.3	p = 0.510

* p<0.05 indicates significant difference between case and control groups

Significent lower hemoglobin levels was observed in patients compared to controls **(Table 2)**. Patients had significently lower plasma BMP-7 concentration than controls **(Table 2).** Both case and controls had Vitamin D plasma concentration less than 30 ng/ml **(Table 2)**. No significant association was observed between BMPs with age, gender, BMI, occupation or drinking black tea **(S1 Table)**. Vitamin D was significantly correlated with age, gender, and occupation **(S1 Table)**.

Of those who completed to follow up 30 were males and 15 females. Their ages ranged from 18 to 65 years (mean = 37.53±12.8 years). Twenty-three patients had upper limb fractures, while 22 had lower limb fractures **(Table 3)**. Twenty were operated and 25 had conservative management. Average time for functional recovery was 4.1 months ± 2.6. There was no significant difference between the upper limb and lower limb recovery time in this study **(Table 3)**.

**Table 3:** Functional outcome of patients with long bone fractures.

	Number of patients	Conservative management	ORIF	Time to functional recovery*
**Upper limb**	23	18	5	4.3 months ± 3.2
**Lower limb**	22	7	15	3.9 months ± 1.9
**Total**	45	25	20	4.1 months ± 2.6

ORIF = open reduction and internal fixation

*p > 0.05 no significant difference between conservative management and ORIF

## Discussion

In this study the patients and controls were well matched in age, gender, BMI and occupation. Hemoglobin level was significantly higher among controls, due to blood loss with fractures [21].

Plasma concentrations of BMP-2 in **(Table 2)** were much lower in this study compared to previously cited (above 500 pg/ml) [6]. Plasma concentration of BMP-7 has been unique as well; it is much lower than observed in another literature, as low as 146pg/ml [6] while it was less than 70pg/ml in this study. This low BMP-2 and BMP-7 cannot be explained, however, other findings on Sudanese subjects displayed similar low bio profile levels such omega-3 [22] which are of course not linked, yet implies that Sudanese subjects might have lower biochemical profiles than the international baseline. Significant difference in BMP-7 plasma concentration was observed between patients and controls. This coincides with previous studies favoring the role BMP-7 acting on healing properties of fractures [23]. Having lower plasma concentrations of BMP-7 among patients could be due to redistribution of BMP-7 in the body to the fracture site to help in the healing process.

Vitamin D insufficiency (20–30 ng/ml) was observed among patients and controls. Previous studies from Sudan have reported similar results [24]. Perhaps, it is the avoidance of sunlight is the explanation for such finding; due to extremely high temperature throughout the day, people would stay most of the day under shades or indoor. Manual laborers had higher plasma Vitamin D than mental laborers due to long Sun-exposure time by manual laborers.

Drinking black tea did not have any significant correlation with plasma vitamin D or plasma BMPs levels. This favors excluding black tea from becoming a negative affecter on BMPs, or Vitamin D. There was no significant association between BMI and BMPs similar to previously reported. BMI had no influence on fracture’s healing.

Forty-five patients had been followed up. Most patients took longer than a month to achieve optimum functional recovery. It was reported that hard callus formation would complete by one month from fracture, essential to resume daily activities [25]. Perhaps, it is due to lower BMPs or Vitamin D that healing took longer than expected. Interestingly, the data support the direct correlation between plasma BMP-7 levels and fracture duration; and the role of BMP-7 in speeding healing rates. BMP-7 levels rising at the end of healing resembles collagen III amino terminal peptide as a marker for healing [26].

## Conclusions

Sudanese subjects have lower BMP-2, BMP-7 and Vitamin D plasma concentrations than non-Sudanese literature reports. Various biometric factors such as age, gender, and BMI had no effect on the basal levels of BMP-2 and BMP-7. BMP-7 is intimately related to fracture physiology; they significantly drop in blood after a fracture.

## Supporting information

S1 File (DOCX)

S1 Table Pearson correlation tests between vitamin D, TGFβ1, BMP-7, and BMP-10 with age, gender, physical activity, BMI, hemoglobin, and black tea. (DOCX)

S2 Table Plasma vitamin D based on gender, age, body mass index (BMI), and occupation. (DOCX)
