# Supplementary material for: Assessment of plasma BMP-2, BMP-7, BMP-10, vitamin D, and TGF β1 in simple fractures among Sudanese patients
Source: PLoS One. 2021 Feb 19;16(2):e0247472. doi: 10.1371/journal.pone.0247472 (PMC7895376; doi:10.1371/journal.pone.0247472)
Supplement: S1 File — (DOCX) [file pone.0247472.s001.docx]

**Data collection**

After volunteers fully understood the purpose of the project and signed, a standard questionnaire was used to collect initial information of each subject: name, age, gender, occupation, and history of previous fracture, treatment, and recovery. Physical examination was also conducted including: general examination, blood pressure measurement while the volunteer was sitting (1), height measured using measure tape with volunteer standing or lying flat on bed. Body mass index (BMI) was calculated from dividing the weight (in kilograms) by the square height (in meters)(2). Random blood sugar was measured using hand held glucometer (GlucoLab)(3,4). Occupation needed a simple self-explanatory classification; either manual labour – this category spend most of their day in outdoor activities, in jobs demanding lifting more than 20 pounds, in addition to, standing for longer periods – such as metal smiths, farmers, and carpenters. On the other hand, some jobs require low physical activates, and less hours standing, even not lifting more than 10 pounds of weight(5,6). Jobs like teachers, office workers, house attendants, and even students can be termed sedentary jobs. Black tea consumption categorized based on previous study(7).

**Blood sample collection and Initial X-ray:**

- After obtaining written consents from the participants.
- A digital camera on the illuminator pictured initial X-ray of the fracture site(8), and then the picture was saved in a personal computer and external hard drive as soft data. Fractures were classified according to AO (Arbeitsgemeinschaft für Osteosynthesefragen) classification of long bone fractures(9).
- After complete disinfection of the arm by alcohol swap, a blood sample was collected from peripheral vein (6 ml) of each patient from 8 am to 12 pm to avoid circadian variation(10), 4 ml in heparin tube and 2 ml in EDTA test tube.
- Blood sample was taken from each control or patient **once** (within 90 days from the onset of fracture).
- CBC was calculated from EDTA tubes(11)(12).
- Both EDTA and Heparin blood sample were centrifuged at 1,000 to 2,000 rpm for 10 minutes to separate plasma from cells(13).
- Plasma from heparin tube was stored at -80^o^C freezer to be used for quantitative enzyme linked immunosorbent assay (ELISA)
- EDTA tubes cells were stored at -20^o^C for DNA extraction

**Sandwich ELISA**

A quantitative technique conducted under standard protocols based on manufacturers’ guidelines(14)(15)**:**

**Vitamin D ELISA**

- Prepared 25-OH Vitamin D ELISA kit from (EUROIMMUN EQ 6411-9601) was used.
- 200 μL of standard, control, and samples (diluted 1:26 in biotin) were added to pre-coated 96 wells – incubated for 120 min at room temperature.
- Wash 3 times by wash buffer.
- 100 μL of enzyme conjugate (streptavidin peroxidase) was added to each well, incubated for 30 min at room temperature.
- Wash 3 times.
- 100 μL of chromogen/substrate was added to each well, incubated in dark room for 15 minutes.
- 100 μL of stop solution was added to each well.
- ELISA reader at wavelength of 450 nm did photometric measurement.
- Standard curve was made and equation formed.
- Samples concentrations were found from the equation.
- Results were recorded.

**BMP-2 ELISA**

- Prepared ELISA kit was used (R&D systems Human BMP-2 DuoSet ELISA Catalog no. DY355-05)
- Coating was done by adding 100 μL of capture antibodies to each well, incubation at room temperature overnight.
- Wash by wash buffer 3 times.
- Blocking.
- Wash by wash buffer 3 times.
- 100 μL of standard (serial dilution) and samples were added to each well, incubated at room temperature for 2 hours.
- Wash by wash buffer 3 times
- 100 μL of detection antibodies was added to each well, incubated at room temperature for 2 hours.
- Wash by wash buffer 3 times.
- 100 μL of working dilution of streptavidin –HRP was added to each well, incubated at dark room for 20 minutes.
- Wash by wash buffer 3 times.
- 100 μL of substrate was added to each well, incubated in dark room for 20 minutes.
- 50 μL of stop solution was added to each well.
- ELISA reader at wavelength of 450 nm did photometric measurement.
- Standard curve was made and equation formed.
- Samples concentrations were found from the equation.
- Results were recorded.

**BMP-7 ELISA**

- Prepared ELISA kit was used (R&D systems Human BMP-7 DuoSet ELISA Catalog no. DY354)
- Coating was done by adding 100 μL of capture antibodies to each well, incubation at room temperature overnight.
- Wash by wash buffer 3 times.
- Blocking.
- Wash by wash buffer 3 times.
- 100 μL of standard (serial dilution) and samples were added to each well, incubated at room temperature for 2 hours.
- Wash by wash buffer 3 times
- 100 μL of detection antibodies was added to each well, incubated at room temperature for 2 hours.
- Wash by wash buffer 3 times.
- 100 μL of working dilution of streptavidin –HRP was added to each well, incubated at dark room for 20 minutes.
- Wash by wash buffer 3 times.
- 100 μL of substrate was added to each well, incubated in dark room for 20 minutes.
- 50 μL of stop solution was added to each well.
- ELISA reader at wavelength of 450 nm did photometric measurement.
- Standard curve was made and equation formed.
- Samples concentrations were found from the equation.
- Results were recorded.

**BMP-10 ELISA**

- Prepared ELISA kit was used (R&D systems Human BMP-10 DuoSet ELISA Catalog no. DY2926-05)
- Coating was done by adding 100 μL of capture antibodies to each well, incubation at room temperature overnight.
- Wash by wash buffer 3 times.
- Blocking.
- Wash by wash buffer 3 times.
- 100 μL of standard (serial dilution) and samples were added to each well, incubated at room temperature for 2 hours.
- Wash by wash buffer 3 times
- 100 μL of detection antibodies was added to each well, incubated at room temperature for 2 hours.
- Wash by wash buffer 3 times.
- 100 μL of working dilution of streptavidin –HRP was added to each well, incubated at dark room for 20 minutes.
- Wash by wash buffer 3 times.
- 100 μL of substrate was added to each well, incubated in dark room for 20 minutes.
- 50 μL of stop solution was added to each well.
- ELISA reader at wavelength of 450 nm did photometric measurement.
- Standard curve was made and equation formed.
- Samples concentrations were found from the equation.
- Results were recorded.

**TGFβ1 ELISA**

- Prepared ELISA kit was used (R&D systems Human TGFβ1 DuoSet ELISA Catalog no. DY240-05)
- Coating was done by adding 100 μL of capture antibodies to each well, incubation at room temperature overnight.
- Samples activation; 40 μL plasma was added to 20 μL 1 N HCL (incubated for 10 minutes at room temperature), then 20 μL of 1.2 N NaOH/0.5 M HEPES was added to the mixture.
- Wash by wash buffer 3 times.
- Blocking.
- Wash by wash buffer 3 times.
- 100 μL of standard (serial dilution) and samples (20-fold dilution) were added to each well, incubated at room temperature for 2 hours.
- Wash by wash buffer 3 times
- 100 μL of detection antibodies was added to each well, incubated at room temperature for 2 hours.
- Wash by wash buffer 3 times.
- 100 μL of working dilution of streptavidin –HRP was added to each well, incubated at dark room for 20 minutes.
- Wash by wash buffer 3 times.
- 100 μL of substrate was added to each well, incubated in dark room for 20 minutes.
- 50 μL of stop solution was added to each well.
- ELISA reader at wavelength of 450 nm did photometric measurement.
- Standard curve was made and equation formed.
- Samples concentrations were found from the equation.
- Results were recorded.

**Long bone fracture healing follow up:**

Series of clinical examinations and radiographic images (in safe and adequate doses)(8,16,17) were conducted at specified follow up sessions. Physical and functional recovery was assessed. Follow up of patients up to point of regaining functional recovery. i.e. For the upper limb function in daily activities such as combing, bathing, lifting, and taking care of hygiene, while for the lower limb; standing, walking unassisted, climbing stairs, and squatting(18).

# References

1. Hamied LIA, Sofiatin Y, Rakhmilla LE, Putripratama AA, Roesli RMA. Comparison of Mercury, Aneroid and Digital Sphygmomanometer in Community Setting. J Hypertens. 2015;

2. Queensland Goverment. Using Body Mass Index. Nutr Educ Mater Online. 2013;

3. Pastakia SD, Ali SM, Kamano JH, Akwanalo CO, Ndege SK, Buckwalter VL, et al. Screening for diabetes and hypertension in a rural low income setting in western Kenya utilizing home-based and community-based strategies. Global Health. 2013;

4. Colagiuri S, Sandbæk A, Carstensen B, Christensen J, Glumer C, Lauritzen T, et al. Comparability of venous and capillary glucose measurements in blood. Diabet Med. 2003;

5. Steeves JA, Tudor-Locke C, Murphy RA, King GA, Fitzhugh EC, Harris TB. Classification of occupational activity categories using accelerometry: NHANES 2003-2004. Int J Behav Nutr Phys Act. 2015;

6. Kuster RP, Huber M, Hirschi S, Siegl W, Baumgartner D, Hagströmer M, et al. Measuring sedentary behavior by means of muscular activity and accelerometry. Sensors (Switzerland). 2018;

7. Chen Z, Pettinger MB, Ritenbaugh C, LaCroix AZ, Robbins J, Caans BJ, et al. Habitual tea consumption and risk of osteoporosis: A prospective study in the women’s health initiative observational cohort. Am J Epidemiol. 2003;

8. Patel DS, Roth M, Kapil N. Stress fractures: Diagnosis, treatment, and prevention. Am Fam Physician. 2011;

9. Meinberg EG, Agel J, Roberts CS, Karam MD, Kellam JF. Fracture and Dislocation Classification Compendium-2018. J Orthop Trauma. 2018;

10. van Baardewijk LJ, van der Ende J, Lissenberg-Thunnissen S, Romijn LM, Hawinkels LJAC, Sier CFM, et al. Circulating bone morphogenetic protein levels and delayed fracture healing. Int Orthop. 2013;

11. George-Gay B, Parker K. Understanding the complete blood count with differential. J Perianesthesia Nurs. 2003;

12. Srinivasan JD, Helwani MA. Complete Blood Count (CBC). In: Data Interpretation in Anesthesia. 2017.

13. Kersaudy-Kerhoas M, Sollier E. Micro-scale blood plasma separation: From acoustophoresis to egg-beaters. Lab on a Chip. 2013.

14. Elmekki MA, Elhassan MM, Ozbak HA, Mukhtar MM. Elevated TGF-beta levels in drug-resistant visceral leishmaniasis. Ann Saudi Med. 2016;

15. Osmekhina E, Neubauer A, Klinzing K, Myllyharju J, Neubauer P. Sandwich ELISA for quantitative detection of human collagen prolyl 4-hydroxylase. Microb Cell Fact. 2010;

16. Radiology Society of North America, American College of Radiology. Patient Safety - Radiation Dose in X-Ray and CT Exams. Radiologyinfo.org. 2016.

17. Eastaugh-Waring SJ, Joslin CC, Hardy JRW, Cunningham JL. Quantification of fracture healing from radiographs using the maximum callus index. In: Clinical Orthopaedics and Related Research. 2009.

18. Nielson CM, Marshall LM, Adams AL, Leblanc ES, Cawthon PM, Ensrud K, et al. BMI and fracture risk in older men: The osteoporotic fractures in men study (MrOS). J Bone Miner Res. 2011;
