# Supplementary material for: Assessment of plasma BMP-2, BMP-7, BMP-10, vitamin D, and TGF β1 in simple fractures among Sudanese patients
Source: PLoS One. 2021 Feb 19;16(2):e0247472. doi: 10.1371/journal.pone.0247472 (PMC7895376; doi:10.1371/journal.pone.0247472)
Supplement: S1 Table — (DOCX) [file pone.0247472.s002.docx]

**S1 Table. Pearson Correlation tests between Vitamin D, TGFβ1, BMP-7, and BMP-10 with Age, gender, physical activity, BMI, hemoglobin, and black tea**

| Variables | Age | Gender | Physical activity | BMI | Hemoglobin | Black Tea |
| --- | --- | --- | --- | --- | --- | --- |
| Vitamin D | p<0.01* | p=0.01* | p=0.005* | p=0.89 | p=0.59 | p=0.25 |
| TGFβ1 | p=0.27 | p=0.85 | p=0.23 | p=0.74 | p=0.008* | p=0.48 |
| BMP-2 | p=0.59 | p=0.81 | p=0.36 | p= 0.69 | p= 0.0.62 | p= 0.57 |
| BMP-7 | p=0.94 | p=0.62 | p=0.1 | p=0.9 | p=0.29 | p=0.3 |
| BMP-10 | p=0.3 | p=0.2 | p=0.1 | p=0.7 | p=0.19 | p=0.81 |

* p value <0.05 indicates significant correlation

BMP: bone morphogenetic protein, BMI: body mass index, TGF: transforming growth factor
