# Supplementary material for: Assessment of plasma BMP-2, BMP-7, BMP-10, vitamin D, and TGF β1 in simple fractures among Sudanese patients
Source: PLoS One. 2021 Feb 19;16(2):e0247472. doi: 10.1371/journal.pone.0247472 (PMC7895376; doi:10.1371/journal.pone.0247472)
Supplement: S2 Table — (DOCX) [file pone.0247472.s003.docx]

**S2 Table. Plasma vitamin D based on gender, age, body mass index (BMI), and occupation**

| Vitamin D (ng/ml) (m±SD) | | |
| --- | --- | --- |
|  | Case | Control |
| Gender |  |  |
| Males | 29.65 ±12.52 | 28.14 ± 10.43 |
| Females | 15.29 ± 9 | 22.34 ± 13.08 |
| Age (years) |  |  |
| ≤ 40 years | 26.5 ±12.61 | 23.6 ± 11.24 |
| > 40 years | 22.8 ± 14.1 | 31.55 ± 10.47 |
| BMI (kg/m^2^) |  |  |
| < 18 | 27.8 ±12.84 | 25.98 ± 4.66 |
| 18 – 25 | 27.52 ±13.65 | 27.37 ± 11.94 |
| 25 – 30 | 21.62 ± 12.91 | 25.95 ± 11.81 |
| > 30 | 18.1 ± 10.64 | 15.25 ± 15.91 |
| Occupation |  |  |
| Mental labor | 22.28 ±13.4 | 24.44 ± 11.33 |
| Manual labor | 31.23 ±11.66 | 31.55 ± 10.76 |

* m= mean, SD= standard deviation
